# Safety of gadobutrol in more than 1,000 pediatric patients: subanalysis of the GARDIAN study, a global multicenter prospective non-interventional study

**DOI:** 10.1007/s00247-016-3599-6

**Published:** 2016-04-04

**Authors:** Katja Glutig, Ravi Bhargava, Gabriele Hahn, Wolfgang Hirsch, Christian Kunze, Hans-Joachim Mentzel, Jürgen F. Schaefer, Winfried Willinek, Petra Palkowitsch, on behalf of the GARDIAN study group

**Affiliations:** 1 Children’s Center Dresden-Friedrichstadt, Fachärztin für Radiologie und Kinderradiologie, Überörtliche Radiologische Gemeinschaftspraxis Dresden-Loschwitz, Standort Kinderzentrum Dresden-Friedrichstadt, Friedrichstr. 32, 01067 Dresden, Germany; 2 Stollery Children’s Hospital, University of Alberta, Edmonton, AB Canada; 3 Pediatric Radiology, University Hospital Carl Gustav Carus Dresden, Dresden, Germany; 4 Pediatric Radiology, University Hospital Leipzig, Leipzig, Germany; 5 Pediatric Radiology, Martin-Luther-University Hospital Halle/Wittenberg, Halle, Germany; 6 Pediatric Radiology, University Hospital Jena, Jena, Germany; 7 Division of Radiology, University Hospital Tuebingen, Tuebingen, Germany; 8 Radiology, University Hospital Bonn, Bonn, Germany; 9 Medical & Clinical Affairs Radiology, Bayer Pharma, Berlin, Germany

**Keywords:** Children, Gadobutrol, Gadolinium-based contrast agent, Magnetic resonance imaging, Magnetic resonance angiography

## Abstract

**Background:**

Gadobutrol is a gadolinium-based contrast agent, uniquely formulated at 1.0 mmol/ml. Although there is extensive safety evidence on the use of gadobutrol in adults, few studies have addressed the safety and tolerability of gadobutrol in pediatric patients.

**Objective:**

This subanalysis of data from the GARDIAN study evaluated the safety and use of gadobutrol in pediatric patients (age <18 years).

**Materials and methods:**

The GARDIAN study was a large phase IV non-interventional prospective multicenter post-authorization safety study performed in Europe, Asia, North America and Africa. A total of 23,708 patients were included who were scheduled to undergo cranial or spinal MRI, liver or kidney MRI, or MR angiography with gadobutrol enhancement. The primary study endpoint was the overall incidence of adverse drug reactions (ADRs) and serious adverse events (SAEs) following gadobutrol administration.

**Results:**

The GARDIAN study included 1,142 children (age <18 years) who received gadobutrol at a mean dose of 0.13 (range 0.04–0.50) mmol/kg body weight. Gadobutrol was well tolerated in these children, with low rates of ADRs (0.5%) and no SAEs, consistent with results in adults enrolled in the GARDIAN study. Rates of adverse events and ADRs were unrelated to pediatric age or gadobutrol weight-adjusted dose. There were no symptoms suggestive of nephrogenic systemic fibrosis. Investigators rated the contrast quality of gadobutrol-enhanced images as good or excellent in 97.8% of pediatric patients, similar to the main study population.

**Conclusion:**

Gadobutrol is very well tolerated and provides excellent contrast quality at the recommended weight-adjusted dose in children (age <18 years), similar to the profile in adults.

## Introduction

Gadobutrol (Gd-DO3A-butrol, Gadovist®, Gadavist®; Bayer Pharma, Leverkusen, Germany) is a second-generation gadolinium-based contrast agent (GBCA) used to enhance tissue contrast in MRI for a range of approved indications in adults and children of all ages [1]. Gadobutrol recently (January 2015) received approval from the U.S. Food and Drug Administration (FDA) for use in MRI of the central nervous system in children younger than 2 years, and gadobutrol is approved in many other countries, including those in the European Union (EU), for whole-body MRI in this age group [2].

Gadobutrol is an extracellular non-ionic macrocyclic paramagnetic contrast agent that is uniquely formulated at 1.0 mmol/ml, i.e. twice the gadolinium concentration of other currently licensed GBCAs. Combined with its high relaxivity, gadobutrol provides the highest T1-shortening effect per milliliter compared with conventional GBCAs, which contributes to an increase in signal intensity [3, 4]. The more compact bolus shape permitted by the higher (1 M) gadolinium concentration is associated with enhanced image quality in faster imaging techniques [5, 6]. The macrocyclic structure of gadobutrol provides greater stability of the chelate and reduced release of gadolinium ions compared with linear GBCAs [7]. Release of gadolinium ions has been associated with the development of nephrogenic systemic fibrosis (NSF) in people with impaired renal function [8, 9]. In view of these characteristics, gadobutrol has been placed by the American College of Radiology, European Medicines Agency (EMA), and the European Society of Urogenital Radiology in the lowest risk category for development of NSF [10–12].

The recommended standard dose of gadobutrol for intravenous injection is 0.1 mmol/kg body weight, with doses up to 0.3 mmol/kg body weight approved for specific indications in adults. At these doses, the efficacy and safety of gadobutrol have been demonstrated in numerous clinical studies in adults [1, 13, 14]. By contrast, there have been few studies of gadobutrol in pediatric patients [1, 15]. In the absence of direct evidence, strategies for use of contrast agents in the pediatric population [16] are typically extrapolated from adult studies.

GARDIAN (Gadovist® in Routine Diagnostic MRI – Administration in Non-selected Patients; NCT01095081) was a prospective large-scale multicenter non-interventional study initiated at the request of the German authority, when the label in the EU was extended to use in pediatric patients. The study was designed to evaluate the safety and tolerability of gadobutrol use in approved indications in people requiring routine contrast-enhanced MRI [17]. The main study results from GARDIAN, excluding the pediatric data, are reported elsewhere [17]. The current subanalysis focuses on children (<18 years), with the primary aim to investigate the incidence of adverse events (AEs), adverse drug reactions (ADRs) and serious adverse events (SAEs) in this population. The contrast quality of gadobutrol was also assessed by the study investigators.

## Materials and methods

GARDIAN was a phase IV non-interventional prospective multicenter post-authorization safety study [17]. In this post-approval commitment, data were planned to be collected on at least 20,000 patients in total, including at least 600 children younger than 18 years and 100 children younger than 8 years. Bayer Pharma provided funding for the study design, data collection, data management and data evaluation of the GARDIAN study and the current study subanalysis.

### Ethical approval

Local ethics committee/institutional review board approval was obtained at each study site. Prior to study enrollment each child or guardian provided written informed consent for the child’s data to be used for scientific purposes, consistent with the ethical principles of the Declaration of Helsinki and the International Conference of Harmonisation guidelines, Good Clinical Practice (ICH-GCP). The study additionally adhered to guidelines of the EMA, the FDA and local laws and regulations.

### Participants

Between August 2010 and April 2013, boys and girls younger than 18 years who were scheduled to undergo approved use of gadobutrol in cranial or spinal MRI, liver or kidney MRI, or MR angiography were recruited from 272 study centers in 17 countries across Europe, Asia, North America and Africa (Table 1). Gadobutrol was administered according to usual clinical practice, with no additional diagnostic or monitoring processes for study purposes. Children with contraindications as stated in the Summary of Product Characteristics for Gadovist (e.g., hypersensitivity to the active substance or to any of the excipients) were excluded. Children with moderate or severe renal impairment (estimated glomerular filtration rate [GFR]: 30–59 or <30 ml/min/1.73 m^2^, respectively) were scheduled for follow-up investigation after 3 months in accordance with routine practice, at the discretion of the treating physician.

**Table 1:** Number of pediatric patients per country (safety population)

Country	Total number of pediatric patients (*n*, % by region)
*Asia*	*425* (*37.2*)
China	156 (36.7)
Kazakhstan & Kyrgyzstan	144 (33.9)
Korea	108 (25.4)
Taiwan	16 (3.8)
Thailand	1 (0.2)
*Europe*	*650* (*56.9*)
Bosnia & Herzegovina	28 (4.3)
Czech Republic	6 (0.9)
France	9 (1.4)
Germany	465 (71.5)
Greece	8 (1.2)
Hungary	2 (0.3)
Italy	17 (2.6)
Russia	101 (15.5)
Spain	14 (2.2)
*Other regions*	*67* (*5.9*)
Canada	63 (94.0)
South Africa	4 (6.0)

### Outcome measures

Patient data (demographics, medical data, safety parameters, treatment signs and symptoms) were documented by the treating physician, nurse or other eligible person during the MR visit. Details on the contrast medium intravenous injection (dose, amount and flow rate) and physician assessment of contrast quality were also recorded.

ADRs and SAEs were the primary study outcomes. The rates, quality and symptoms of AEs and ADRs were recorded over approximately 1 day from the administration of gadobutrol and were coded using the standardized *Medical Dictionary for Regulatory Activities* (*MedDRA*, version 13.0). AEs were defined as any undesirable outcomes that occurred during or after administration of gadobutrol, while ADRs were AEs that were considered by the treating physician to be attributable to gadobutrol administration. The categorization and severity of AEs and ADRs were based on the judgment of the treating physician. Secondary safety outcomes included ADR rates analyzed by the gadobutrol dose administered and by medical indication.

To monitor patients at high risk for NSF in this observational trial, a set of questions was provided to investigators to assess the children’s precise renal conditions. Any skin reactions suggestive of NSF were compulsorily followed up by investigators as AEs.

The image quality was rated subjectively by investigators on a four-point scale (poor, moderate, good or excellent).

### Statistical analyses

Descriptive analyses were performed on non-missing continuous (median, mean, standard deviation, maxima and minima, upper and lower quartiles) and categorical data (frequency tables).

Event rates for AEs and ADRs in children were calculated as a proportion of the pediatric study population. All enrolled subjects who were administered at least 1 dose of gadobutrol were included in the safety population. Only patients who underwent an MRI/MR angiography scan were included in the efficacy population. Statistical analyses were performed using SAS version 9.2 (SAS Institute, Cary, NC).

## Results

### Baseline demographics

A total of 23,708 patients in the main GARDIAN study were administered at least 1 unit of gadobutrol [17]. Of these, 4.8% (*n* = 1,142) were pediatric patients younger than 18 years. Pediatric patients were mainly enrolled from Europe (56.9%), followed by Asia (37.2%) and other regions (5.9%) (Table 1). Most children were in the age range of 7 years to younger than 18 years (84.9%), while 14.7% were ages 2 years to younger than 7 years, and 0.4% were younger than 2 years (Table 2).

**Table 2:** Demographics and baseline characteristics of the pediatric safety population

Demographic data		*N* (%) pediatric patients/median values
Total patients		1,142
Gender (*n*, %)	Male	604 (52.9)
	Female	538 (47.1)
Age groups (*n* [%], years)	<18 years (total)	1,142 (100)
	<2 years	4 (0.4)
	2 – <7 years	168 (14.7)
	7 – <18 years	970 (84.9)
Body weight (median, kg)	<18 years (total)	45.0
	<2 years	9.5
	2 – <7 years	18.5
	7 – <18 years	50.0
BMI (median, kg/m^2^)	<18 years (total)	19.0
	<2 years	19.1
	2 – <7 years	15.7
	7 – <18 years	19.7
At least 1 contrast medium risk factor (*n* [% of age subgroup])	<18 years (total)	95 (8.3)
	<2 years	0
	2 – <7 years	12 (7.1)
	7 – <18 years	83 (8.6)
Types of risk for contrast medium reaction (*n*, %)	Allergy	81 (7.1)
	Bronchial/asthma	25 (2.2)
	Previous contrast medium reaction	1 (0.1)

*BMI* body mass index

Risk factors for contrast medium reaction in children included allergy (*n* = 81), bronchial asthma (*n* = 25) and history of contrast medium reaction (*n* = 1). Concomitant cardiac conditions in the pediatric population included systemic arterial hypertension (*n* = 6), heart failure (*n* = 3), cardiac arrhythmia (*n* = 2) and coronary heart disease (*n* = 2). No children with severe renal impairment were included; six children with moderate renal impairment (GFR 30–59 ml/min/1.73 m^2^) were included. Acute renal failure was reported in one child (age 12.4 years) and no child was on dialysis.

### Gadobutrol dose details

The mean gadobutrol dose delivered was 0.13 mmol/kg body weight, with a range of ≤0.1 mmol/kg in 510 children (44.7%), >0.1–0.2 mmol/kg in 551 (48.3%), >0.2–0.3 mmol/kg in 67 (5.9%) and >0.3 mmol/kg body weight (i.e. above the approved dose) in 14 (1.2%) (Table 3). Mean gadobutrol doses categorized by pediatric age group were 0.21 (range 0.11–0.33) mmol/kg for <2 years, 0.13 (0.06–0.50) mmol/kg for 2 years to <7 years and 0.13 (0.04–0.36) mmol/kg for 7 years to <18 years.

**Table 3:** Dose groups in the pediatric safety population

Dose group (mmol/kg body weight)	<2 years, *n* (%)	2 – <7 years, *n* (%)	7 – <18 years, *n* (%)	Total, *N* (%)
≤0.1	0	88 (52.4)	422 (43.5)	510 (44.7)
>0.1 – 0.2	3 (75.0)	64 (38.1)	484 (49.9)	551 (48.3)
>0.2 – 0.3	0	10 (6.0)	57 (5.9)	67 (5.9)
>0.3^a^	1 (25.0)	6 (3.6)	7 (0.7)	14 (1.2)
Total	4 (100)	168 (100)	970 (100)	1,142 (100)

^a^Above the approved dose

### Adverse events (primary endpoint)

AEs were reported in 8 of the 1,142 (0.7%) children in the safety population. Individual AEs included vomiting (*n* = 3, 0.26%), nausea (*n* = 2, 0.18%), and injection-site reaction, urticaria, dyspnea and eyelid edema (*n* = 1 each, 0.09%); one child had two AEs (nausea and vomiting). The severity and relationship of the AEs are described in Table 4. Five children experienced AEs of mild maximum intensity, while the remaining AEs were of moderate maximum intensity. No AEs were rated severe or serious. Complete recovery was achieved in all eight children who reported an AE, with no action required for the majority of these events (66.7%). One AE (eyelid edema) persisted for 5 h and was treated pharmacologically, and another two AEs (vomiting and nausea) received other treatments and did not extend beyond 5 min in duration. Among the children with risk factors for contrast medium reaction, one child in the 2- to <7-year range with bronchial asthma experienced vomiting, which was assessed as unrelated to the study drug. Another child, in the 7- to <18-year age group, with concomitant allergy, experienced eyelid edema, which was assessed as related to the study drug.

**Table 4:** Adverse event (*AE*) and adverse drug reaction (*ADR*) details per child in the pediatric safety population

Patient (gender/age [years])	AE/ADR	Dose (mmol gadolinium/kg body weight)	Relationship with drug	Severity
Female/17	Nausea and vomiting	0.11	Yes	Mild
Male/6	Vomiting	0.13	Yes	Moderate
Male/13	Nausea	0.11	Yes	Mild
Female/15	Eyelid edema	0.10	Yes	Moderate
Female/5	Urticaria	0.11	Yes	Moderate
Female/14	Injection-site reaction	0.11	Yes	Mild
Female/5	Vomiting after 15 min	0.10	No	Mild
Male/13	Dyspnea after 24 h	0.10	No	Mild

Of the eight children with AEs, six were classified with an ADR (0.5% of the 1,142 pediatric population) (Table 4). No association was observed between the gadobutrol dose and the frequency of AEs or ADRs (Table 3).

No skin reactions suggestive of NSF were reported in any child in this study.

### MRI examinations and contrast medium injection

The mean injection rate in children was 1.45 ml/s (range 0.00–5.00 ml/s), with an automatic injector used in 20.9%. The magnetic field strength used was 1.5 T in most cases (63.4%), followed by 3 T (17.0%), 1 T (12.9%) or other strengths (6.8%). Most investigations in the pediatric population were for suspected tumors (52.9%), followed by inflammatory disease (13.8%), tumor staging (5.6%), trauma (5.2%), infarction (1.9%) and multiple sclerosis (1.8%), with other indications in the remainder (18.8%). The brain and spine were investigated in 77.7% of patients, the vessels using MR angiography in 19.4%, kidneys in 1.6%, liver in 1.2% and others in 0.1%.

The contrast quality of gadobutrol-enhanced images was evaluated subjectively by physicians as excellent in 63.7%, good in 34.0%, moderate in 2.2% and poor in 0.1% of the children.

## Discussion

GARDIAN is a non-interventional MRI study that has provided the largest analysis of safety and tolerability for the routine use of gadobutrol in clinical practice. As observed in adults enrolled in GARDIAN [17], gadobutrol was very well tolerated in children, with low rates of AEs (0.7%) and ADRs (0.5%), and no SAEs. Rates of AEs and ADRs were unrelated to gadobutrol weight-adjusted dose and age in the pediatric population. The contrast quality of gadobutrol-enhanced images was good or excellent in almost all children (97.8%), similar to the main study population [17].

The incidence of ADRs in children was slightly lower than in the main GARDIAN study (0.5% vs. 0.7%). This is consistent with a lower rate of contrast medium reactions in children compared with adults in the large retrospective analyses by Dillman et al. [18] and Davenport et al. [19]. A prospective open-label study of gadobutrol conducted by Hahn et al. [15] in 138 children ages 2–17 years reported an ADR incidence of 5.8% during a 1-week follow-up, with the lowest rates reported in the younger age group (2–6 years, 2.2%). The higher ADR incidence in the Hahn et al. [15] study compared with the current observational study may be attributed to its design as a controlled phase I/III study that included a longer follow-up duration, pre-defined in the study protocol. In an observational study like the GARDIAN study, mostly acute reactions are captured because no additional interventions, e.g., laboratory value analyses, are possible to uncover other potential AEs. It can be assumed that the ADR rate of gadobutrol in the GARDIAN study reflects the rate of allergy-like reactions. This contributes toward the lower rate of AEs in children in general and as compared to randomized clinical trials [15, 20]. Consistent with the current study, low AE rates have been reported for other GBCAs including gadodiamide, gadopentetate dimeglumine, gadobenate, dimeglumine, gadoteridol and gadoversetamide in children [19, 21–25].

All ADRs reported in children in the GARDIAN study were of mild to moderate intensity, consistent with Hahn et al.’s [15] findings. Vomiting and nausea were the most common ADRs, as is reported in the adult literature on GBCAs [17, 26]. The GARDIAN study reported no NSF-related signs or symptoms in either children or adults.

Limitations of the GARDIAN study include the potential for under-reporting of AEs or ADRs, especially those of mild severity, which is a feature of observational studies. Lack of long-term follow-up in all children, low numbers of children younger than 2 years and the use of a subjective evaluation of image quality by a heterogeneous group of reviewers are additional limitations. The advantages of GARDIAN are its large sample size that included 1,142 children (age <18 years). In addition, the inclusion of a large number of centers from various geographic regions helps overcome the limitations of bias encountered in AE reports from single-center, single-indication studies and increases the representativeness of the results. Finally, the non-interventional design of this study reflects the use of gadobutrol in contemporary practice.

## Conclusion

The results of this large pediatric subanalysis confirm that gadobutrol is a very well-tolerated GBCA that provides excellent contrast quality at the recommended weight-adjusted dose (0.1 mmol/kg body weight) in children (age <18 years), similar to the outcomes in adults enrolled in GARDIAN. Low rates of AEs and ADRs and no SAEs related to gadobutrol were reported in these children. Gadobutrol is concluded to be an appropriate contrast agent for imaging of children in routine clinical practice, requiring no further dose adjustment according to age.
